# Experiences of integrated care: reflections on tensions of size, scale and perspective between ethnography and evaluation

**DOI:** 10.1080/13648470.2018.1507105

**Published:** 2019-02-04

**Authors:** Gemma Hughes

**Affiliations:** Nuffield Department of Primary Care Health Sciences, University of Oxford, Oxford, UK

**Keywords:** Chronic conditions, ethnography, evaluation, integrated care, narrative, phenomenology, temporality

## Abstract

An in-depth case study of integrated health and social care provides the empirical basis for this exploration of tensions between ethnography and evaluation. The case study, developed from a two year period of fieldwork, is based on ethnographic data of individuals’ experiences of living with multiple long-term conditions, their experiences of integrated care, and integrated care commissioning practices. Narrative and phenomenological analysis show how temporal aspects of ethnographic fieldwork contribute to producing knowledge of patients’ experiences. However, tensions emerge when attempting to bring learning from these experiences into discussions about evaluations of services. Data generated from fieldwork are seen as both too ‘big’, in terms of quantity of details, and too ‘small’, in terms of generalisability. Scale is also of concern, as tensions between ethnography and evaluation play out in questions of relevance. Ethnography foregrounds embodied, day-to-day lived experience, bringing the minutiae of daily life into sharp focus whereas evaluators need a wider angle to foreground larger objects of interest; organisations, budgets, services. A further source of tension between ethnography and evaluation emerges in defining interventions as distinct from context, when the conceptual boundary required to distinguish the shape of the intervention within a social world blurs and dissolves under the close gaze of an immersed ethnographer, problematizing attempts to inform causation. Concerns are raised that without greater dialogue about the nature of knowledge produced by patients’ experiences, these experiences are at risk of being marginalised and de-centred.

## Introduction

An in-depth study of integrated health and social care in an urban area of England provides the empirical basis for this paper, which explores tensions arising from attempts to share and mobilise ethnographically derived knowledge of patients’ experiences. I open the paper by introducing the integrated care policy discourse and explaining my methodology for researching a case of the practice of integrated care. Drawing on a vignette of one couple living with multiple chronic conditions, and subject to efforts to integrate care in the case, I explain how I come to know their experiences through narrative and phenomenological interpretations, with attention to the temporal aspects of knowledge production in fieldwork. Tensions encountered in sharing this knowledge in the site lead to an exploration of the ways in which patients’ experiences are more typically represented in service evaluations and other public documents.

Integrated care describes efforts to bridge diverse gaps, including those between legislative and institutional divides between the NHS and social services in the UK; organisational demarcations of service provision; and the boundaries of professional specialisation. Concerns with how to best organise health and social care services to manage these divides, and to contain costs, have shaped the history of health policy in the UK, resulting in continual processes of reform and organisational change. The emergence of the discourse of integrated care found in current health policy stems from these perennial concerns, overlaid with changing ideas about shifting care from hospitals into the community, and interwoven with a growing awareness of the need for chronic rather than acute care inspired by North American models of efficiency. The resulting discourse embeds integrated care in UK health policy as a solution to a range of problems associated with increasing costs, fragmentation and complexity. Integrated care offers the possibility of ‘person-centred coordinated care’ by organising services around individual patients’ needs (*A Narrative for Person-centred Co-ordinated Care*, 2013). The expectation is that by providing more co-ordinated care patients will have better experiences, and they will enjoy improved health which will reduce their need for health services, and so reduce costs. These aspirations, known as the ‘triple aim’ are incorporated into NHS policy as ‘improved health and wellbeing, transformed quality of care delivery, and sustainable finances’ (Berwick, Nolan, and Whittington, 2008, *NHS Five Year Forward View*, 2014).

Integrated care is not a single intervention or solution for a defined cohort, rather it is a term with multiple definitions aimed at diverse populations (Armitage et al., 2009). It is intended to produce outcomes related to the varying facets of improving experience, health and efficiency. Integrated care is enacted in multiple, inter-related ways, conceptualised as taking place at different ‘levels’ within a system, namely: micro (concerned with clinical integration), meso (organisational integration) and macro (system integration) (Valentijn et al., 2013). Evaluating the impact of such activities, whether on patients’ experiences, economic impact or health outcomes raises methodological challenges, as does comparing evaluations, and conducting systematic reviews.

Integrated care is mobilised in NHS health policy targets and outcome measures that set out ambitions to reduce emergency hospital admissions (*Better Care Fund: Policy Framework*, 2014, *The Mandate: A mandate from the government to NHS England: April 2015 to March 2016)* and in pilot programmes intended to test out ways of integrating care: such as the Evercare pilot of case management (Boaden et al., 2005; Gravelle et al., 2007; Sheaff et al., 2009), the Department of Health Integrated Care Pilots (Ling et al., 2012; Roland et al., 2012) and the ongoing English Integrated Care Pioneer Programme (Erens et al., 2017; Eyre, George, and Marshall, 2015; Curry et al., 2013). Evaluations of these programmes use mixed methods to assess impact on use of health services, outcomes, cost, professionals’ views, and patients’ experiences. To date, despite considerable political and organisational support, evaluations have only reported limited evidence of effectiveness. Expectations of integrated care appear optimistic in light of this evidence, (Damery, Flanagan, and Combes, 2016) although the policy discourse of breaking down organisational barriers to create new models of integrated care continues (Hughes, 2017).

I started to investigate the case of integrated care when working for the NHS, expected to ‘do’ integrated care, to ensure cost savings were realised and develop partnerships with local authorities. Contradictions between projections of cost savings resulting from integrated care, and the evaluations of English integrated care programmes cited above, generated questions about the practice and experience of integrated care, which I pursued in my doctoral research, undertaken alongside my NHS commissioning role.

## Methodology: An ethnographic case study of the practice and experience of integrated care

I developed an in-depth case study to examine the practice and experience of integrated care, informed by a tradition of case study inquiry into issues within their social and cultural context (Stake, 1995; Simons, 2009; Burawoy, 2009; Flyvbjerg, 2006). I constructed the case as a ‘bounded system’ (Stake 1995), taking the health planning unit (a sub-region of the city) as the boundaries of the case, and the field. I had prior knowledge of the case from my employment as an NHS commissioner. During fieldwork undertaken from 2014 to 2016, I became participant-observer in my own work as a commissioner and in sharing the social worlds of people living with multiple long term conditions. I followed three interconnected lines of inquiry throughout fieldwork, broadly mirroring the different conceptual levels of integrated care; the experience of individuals; organisational practices; and the macro policy discourse, ‘zooming in’ on lived experiences and ‘zooming out’ (Nicolini, 2009) to practices of integrated care. While I pursue additional lines of inquiry in my doctoral thesis about the commissioning and policy practices of integrated care, the focus of this paper is the experience of patients, and the challenges I encountered in synthesising and sharing these experiences.

My analysis is informed by the phenomenological approach used by Greenhalgh et al. (2013) when they asked: ‘What matters to people with assisted living needs?’. I asked: ‘what matters to people (with integrated care needs) and how does their lived experience affect their use of integrated care?’ From this starting point of a concern with ‘what matters’ to people, I took an inductive approach to analysing interactions with research participants, influenced by both the constructivist grounded theory approach of Charmaz (1991, 2014), and her study of chronic illness. I adopted several grounded theory strategies; iterative data collection and analysis; comparative methods across patient cases; and inductive analysis, though I do not aspire here to the grounded theorist’s goal of constructing theory. In making sense of the experiences I shared with these people living with multiple chronic conditions, many of whom were old, and some of whom were in the last months of their lives, I drew on phenomenological and narrative interpretations.

A phenomenological approach enables inquiry into experiences of everyday embodied practices as ‘fact(s) about the world’ (Pickard and Rogers, 2012) and avoids the reductionism that would discount the day-to-day experience of research participants as being simply caused by their various chronic conditions. Instead these facts are central to understanding how living with chronic conditions changes being in the world (Carel, 2013). In addition to recognising the embodied experiences of research participants through a phenomenological lens, I also draw on narrative methods (Riessman, 2008) in constructing and analysing the meaning that is created from people’s talk as they trace the histories of their conditions and their interactions with services. The tradition of narrative in health and medicine spans individual clinical case studies, personal accounts of illness as well as organisational case studies and policy discourse (Greenhalgh, 2016). Narrative ‘data’, or the stories people tell and live, are important forms of evidence in sharing experiences of illness. Narrative research also provides interpretive methods, exploring how people make sense of, and construct meaning from, their lives. A further extension of narrative research is provided by Mattingly, in her discussion of therapeutic ‘emplotment’ which elucidates the role of narrative in the creation of experience (Mattingly, 1998). My attempt to describe and understand people’s experiences involved observing and understanding their embodied reality, how this was told, and how this interacted with their personal biographies and illness narratives, adopting a person-centred, ‘experience-near’ attention to embodied routines of daily life, described by Mattingly as narrative phenomenology (Mattingly, 2010).

Participant observation in the field enabled me to develop in-depth knowledge of the particular and embodied living circumstances of research participants. My intention was to bring this knowledge into discussions in the site to inform service improvements and evaluations. I sought to connect the discourse and practice of integrated care with the lived experience. The difficulties in connecting the fine-grained experiences encountered through ethnography with the ways of thinking about, and evaluating, interventions to improve health and social care led to the concerns that I explore in this paper as I found the knowledge I had produced did not appear relevant. The detail of peoples’ lives, which mattered so much to my understanding of them, didn’t seem to matter in discussions about services. This paper explores ways in which this knowledge matters, and ways in which it doesn’t. I became concerned not just with understanding what mattered to people, but how these understandings, and therefore these people, mattered.

## Method

My position during fieldwork was dual, I was participant -turned-observer, a health service commissioner embarking on doctoral research into her own organisation’s attempts to improve services, hit targets, manage NHS funds and somehow stay afloat amidst legislative changes and increasing austerity in public sector funding. I was already immersed in the practices of commissioning in the study, so had a particular kind of access to the social world of commissioning and was able to draw on my own experiences in an auto-ethnographic sense. I also set out to understand the phenomena of integrated care from the vantage point of people who were the subjects of these efforts. At the beginning of my fieldwork, integrated case management was being ‘rolled out’ across the area as a way of reducing emergency hospital admissions, and provided the focus for my research. Several hundred people in the case were identified by a combination of algorithmic analysis of routine data and clinical judgement as being at high risk of hospital admission and therefore were enrolled onto the integrated case management caseload. They were assessed by a multi-disciplinary team, assigned a case manager (an expert community nurse) and their care detailed in electronic care plans. I recruited a purposive maximum variety sample of twenty of these people as research participants. Following introductions by their community nurses, I was invited into their homes. There, I listened to their stories, saw how they lived, and who they relied on. I spent hours with people over the months of my fieldwork, getting a glimpse into the long years of their lives. Frequency and duration of contact with each research participant varied according to our relationship and their preferences; with between one and sixteen interviews or visits for each participant (total of 94 interviews/visits) and multiple phone conversations over periods of engagement that lasted between four and nineteen months. During this period, a number of other integrated care initiatives were established in the site, indirectly affecting these research participants. In addition to participant-observation with ‘patient’ research participants, I collated extensive fieldnotes and documents from participant-observation and auto-ethnography of commissioning practices. To explore in detail the concerns of this paper – how peoples’ experiences matter – I focus on two research participants and a selection of documents from the case, an overview of which is provided below.

## The situated case

The case is situated across a geographical area that reaches from the edges of an inner-city to the surrounding suburbs, a health planning unit comprising three city councils with a population of approximately 750,000 people. Parts of the area are deprived, with post-war decline of heavy industry affecting employment and housing provision, other parts are relatively affluent. Health and social care services are under pressure. The local acute NHS hospital trust has a history of quality and financial problems. Radical approaches are being adopted by local councils to create balanced forecast budgets, including significant cuts to services, and rapid regeneration efforts. Integrated health and social care has been pursued in this area for more than fifteen years, activities at different levels include: implementation of case management for people considered to be at high risk of hospital admission; joint commissioning between the NHS and local authorities; and development of strategies to manage population health budgets. Integrated care was inserted into NHS strategic plans as a way of reducing costs; featuring in Quality Innovation Productivity and Prevention (QIPP) schemes as a way of managing financial pressures on the health service (*Long Term Conditions Compendium of Information: Third Edition.*, 2012). During fieldwork, new initiatives included the establishment of an alternative integrated care service (a specialist health practice for people with complex needs), changes to intermediate care services from a bed-based to a community-based service, and an application to devolve budgets from central government to the local health system, reflecting the continual processes of reform and organisational change referred to previously.

## Lived experiences and narratives of chronic conditions and integrated care

People most in need of integrated care have multiple health and social care needs. People with these multi-morbidities are often (though not always) older, likely to have co-morbid physical and mental health difficulties and to live in areas of deprivation (Barnett et al., 2012). A range of concurrent ‘social’ needs arise from living with multiple chronic conditions, especially, though not exclusively in later life, and towards the end of people’s lives. The recursive nature of chronic conditions is associated with recursive use of health and social care services (Manderson and Warren, 2016). Through participant- observation, I aimed to pay close attention to the way in which people lived with multiple chronic conditions, to listen to how they made sense of their conditions in order to understand the things that mattered to them and how they experienced services intended to integrate their care. I have chosen here to examine the experiences of one couple because of the instructive nature of re-interpretations of their situation, and the ways in which they were directly and indirectly affected by integrated care interventions. The vignette that follows is drawn from fieldnotes and recordings of conversations with the pseudonymised Doris and Walt from a 12 month period from 2014 to 2015.

I first meet Doris and Walt on a cold but sunny winter day, when I drive to their home in a quiet, residential area in the suburbs of the city. They live in a purpose-built flat, one of several small, neat blocks built around central gardens and courtyards. My first impressions are that this is well-designed accommodation for older people, with on-site support, attractive buildings, and company close to hand. But Doris explains that she can no longer tend to the flowers in the large plant pot outside their front door, as she can’t use the stairlift down from their first-floor flat unaided. The couple, now 83 and 94 years old, married late in life after meeting at a dance class. They spend their days now in their comfortable living room, communicating via intercom with the manager of the housing block who checks on them every other day or so. Doris buzzes me in, waiting at the top of the stairs to greet me. Even if she could manage the stairs, there is no room for more than one person to be in the small space by the front door. I hear a story later about how this configuration of door and stairs created an impassable bottleneck when an ambulance crew tried to carry Walt out once when he was seriously ill. There wasn’t enough space for the two ambulance crew members, carrying Walt, to open the door inwards. The door had to be removed from its hinges, and now opens outwards.

The aesthetically pleasing material environment, with well-maintained gardens and buildings, outdoor seating and plant pots, seems at first carefully constructed for the enjoyment and comfort of the elderly residents. Privacy and independence behind a front door is close to support from a housing manager and the company of other people in similar circumstances. However, as I listen to the story of Walt’s ambulance trip, jolt at the sound of the communal fire alarm interrupting a peaceful morning, and realise the extent of the restrictions on the couple’s mobility, I develop new interpretations about the life they lead. Being there for extended periods of time contributes to a deeper kind of knowledge than a superficial categorisation of their accommodation. My initial impressions of their comfortable, suitable, home change as their narratives unfold, and I develop a phenomenological understanding of their embodied lives.

Walt describes himself as ‘not very frisky’, in a dry understatement, as he moves slowly and carefully around the small living space. I find out that he rarely lies down to sleep at nights because of his respiratory problems, remaining propped up so he can breathe more easily. Recurring chest and urinary tract infections tire him too, and he no longer eats well. As we sit in their comfortable living room I admire the view over the open green opposite. Doris lowers the blinds a little to keep the sun out of our eyes as we notice the dog walkers and joggers pass by. A flock of geese sit peacefully on the grass. However, this pleasant view pales in comparison to the wide-open spaces of the fens that feature in the photographs Doris later shows me. They used to watch the sun set over the flat fields stretching to the horizon from the garden of their old home. I flick through albums of wedding photos, holidays, Doris’s beloved cat, and van, and their dancing outfits. In the present, I realise that they are virtually unable to leave this flat. Walt hasn’t been out, other than his trip to hospital, for more than 2 years and since Doris fell and injured herself whilst out shopping months ago she can only bring herself to leave the flat with considerable pre-planning and the support of their paid carer, Tracey.

Walt’s demeanour tells me that he doesn’t expect to be particularly ‘frisky’ at the age of 94, his expectations about what he might be able to do, and how he might feel, are modest. Although he is not someone to complain about his health, I observe that he is rarely in a state of bodily comfort or ease and that even his modest hopes of activity are dashed by his continued containment within his home. Further, the contrast between the perfectly adequate home the couple share and what they left behind when they had to leave their rural dwelling, is slowly constructed from photographs, talk and my presence in their home. My initial impressions of comfort and companionship develop into a more complex appreciation of their social isolation. Their comfortable-looking flat is in fact the limits of their physical world, and their slow progression around it is marked by pain and breathlessness.

Doris can no longer reach to wash her own feet. She has previously joked to me about how she struggles to get her socks on in the mornings, but on this occasion her voice is sombre. She explains how she tried to deal with this frustrating situation by dropping a wet sponge onto the shower cubicle floor then treading on it, but not only did this process fail to get her feet properly clean, she found she couldn’t thoroughly dry them afterwards. Doris has had to deal with many serious health problems, having recovered from cancer and experiencing severe pain of arthritis, as well as worrying about Walt’s failing health, yet it is recounting this seemingly trivial detail of not being able to keep parts of her own body clean that causes her to break down in tears. Doris describes herself as a ‘carer, a giver not a receiver’, a role that she has spent many years fulfilling as becomes apparent when she tells me on another occasion about her first husband who she nursed through disability and dementia for 13 years before he died. Doris tries very hard to stay cheerful in the face of difficulties, she talks about putting on her ‘clown’s face’ to try and keep depression at bay and enjoys telling silly jokes, so her fleeting admission of frustration and despair is highly significant.

The combination of a lived body approach that recognises the physicality of being unable to bend a stiff body enough to reach its own extremities, and a narrative approach that comprehends the construction of an identify as a caring wife, explains the depth of despair Doris experiences as she shares her distress of being unable to wash her feet, and how Doris’s lived, bodily experience is inseparable from her identity. This mundane struggle is telling of the social isolation and physical discomfort Doris regularly experiences and the deficit of care available.

With no children, and few relatives, Doris and Walt have a social world as small as their physical world. Tracey, their directly employed carer, visits them three times a week to assist with bathing and housework; they look forward to the conversation and news she brings, and have developed a close relationship – more like a family member than an employee. They have chosen this arrangement rather than one organised by social services to avoid the continual change of personnel that they know this would involve. Doris had a temporary ‘care package’ arranged by hospital social workers after her hip replacement, counting up to 16 different people visiting her before she asked them not to come any more. Tracey, on the other hand, has been a regular, joyful presence over the years. She fits this work around looking after her disabled son, doing any jobs she is asked to, but also brings Christmas decorations, sings as she bustles around the flat, pauses to chat, and arranges for her friend to pop in and check on them when she is on holiday.

Doris’s face lights up when she talks about Tracey, and I am shocked when I hear the news that she is planning to move away when her husband retires. Doris and Walt will lose their beloved supporter. They had already experienced loss earlier in the year when their community matron, Diane, moved to another job. Diane had been allocated to provide integrated case management to the couple and although she was too busy to spend much time with them, they became very fond of her and appreciated her professionalism and caring nature – Doris said she really ‘knew them’. I find that Diane has been recruited to the new specialist health practice, the latest integrated care intervention to be piloted and evaluated in the study site.

During fieldwork I become implicated in Doris and Walt’s narrative. I too, can hardly imagine Tracey not being around for them, so central is she to their lives, yet I can see the unfolding of this other overlapping narrative; Tracey’s story, shaped by her own evolving family dynamics. I connect Doris and Walt’s experience with the health system changes, as I see one integrated care intervention (the provision of integrated case management from Diane) overlapping with another, the establishment of the new service. I receive news of Diane’s move as somewhat inevitable, an experienced nurse will undoubtedly be attracted to, and valued by, a new service. I understand this change as both an unintended consequence of establishing a new initiative, intended to provide better care to people like Doris and Walt, and a personal loss to a lonely couple. I also notice the disconnect between what matters to this couple and the ‘system’ aims, efforts to integrate care focus on avoiding emergency hospital admissions, yet the most disruptive hospital admission occurring during fieldwork is Doris’s elective admission for a hip replacement, an unavoidable event.

## Temporal aspects of ethnographic fieldwork

There are temporal dimensions of fieldwork and analysis that are relevant to the way I generate and report my understanding of Doris and Walt’s lives and experiences. I spend many hours in conversation with them, over a period of a year. This amount of time is a pre-requisite to learning about their experience, to develop this ‘way of knowing’ (Pink, 2011). Although this time commitment is onerous and generates a large quantity of materials in the form of fieldnotes, audio-recordings and transcripts, it remains only a partial connection with Doris and Walt’s lived experience during this time, which in turn is only a small proportion of their lives. While a phenomenological analysis allows me to connect with moments of lived experience at particular points in time, such as the moment when Doris can’t bend to wash her feet, a narrative approach allows these fragments of time to be brought together, and to stretch the field temporally, into the past (as Doris reminisces and shows me old photographs), and into the future (when we try to imagine how they will cope without their familiar carer).

The length of time spent in the field also allows for a historical understanding to develop of different interventions, new services are established, people change jobs, but the relationships with participants continue. Whereas fieldnotes and chronologies, the tools of ethnography, simply elongate, narratives are re-interpreted as time passes. Different narratives emerge over time. In Doris and Walt’s narrative, as new events unfold, past events are reinterpreted. Then there is my narrative, of my own development as a researcher, participating in the metanoia – the series of transformations – that the practice of ethnography can produce (Ingold, 2014). Finally, there is the research narrative, the process of developing an understanding of what is going on here, how people and events are connected, and are changing. These multiple narratives go beyond the linear and chronological understanding of events, and instead allow for re-reading and re-interpretation.

Through ethnography, different kinds of data enable different ‘ways of knowing’. Whereas narrative evokes the form of an arc, encompassing sequences of events across time; phenomenological understandings are, perhaps, more akin to momentary glimpses that illuminate the embodied experience of a particular material world. These moments arose in fieldwork, often in unexpected ways, when a connection was made between my world, and that of the informant, felt as a moment of understanding, a strong feeling of empathy. The longer the period of time spent in participant-observation, the more of these moments that are likely to happen. The development of narratives, and a phenomenological appreciation of experiences narrated, contributes to different ways of knowing through ethnography. The knowledge I have co-produced with Doris and Walt comprises facts about their world, not simply their perspective on the world as I experience it, but an understanding of their experience of being in their world, constrained by their bodily sensations, rendered full of meaning by their individual and shared biographies, and constructed in our shared narrative. This form of knowledge is both instructive, teaching me about what matters to Doris and Walt, and transformative, making them matter to me.

## Tensions of size, scale, and perspective

As part of my embedded and dual role, I aspired to share the knowledge gained about people’s experiences with others in the site to inform efforts to improve integrated care. I represented patients’ experiences in different kinds of texts: case summaries, narratives, and ethnographic portraits. I encountered tensions of size, scale and perspective as I tried to make this knowledge matter in the world of evaluations and decision-making about health services.

Data, in the form of fieldnotes, audio recordings, transcripts and photographs, generated through participant-observation were seen as both too ‘big’ in terms of quantity and too ‘small’ in terms of generalisability. The lengthy material gathered through fieldwork didn’t fit into the packed agendas of formal meetings, there was never enough time to discuss the lengthy histories and detailed situations. I synthesised patients’ narratives into case summaries which proved of interest to local clinicians who recognised the portrayals of interacting health conditions and the complexity of the response required. They nod with recognition as they read about Doris and Walt, and wonder if this material can be used to inform training of clinical staff, helping to engender empathy for their patients, and an understanding of how to build rapport. However, when these experiences are brought into discussions about the future of services, they become too small. Experiences of only a few people, however thickly described, are not appropriately representative, and cannot answer questions about the spread of specific interventions, or the availability of services, across the local population. Careful representation of how patients’ experiences are shaped by their narratives, social context and embodied experiences is not relevant to questions about standard compliance with guidelines and appropriateness of referrals. In short, ethnography produced too much data about too few people to be of great interest to those charged with improving services and making decisions.

A crucial component of participant-observation is to follow what is important to research participants, finding out what is ‘at stake’ for them (Mattingly, 2010). An ethnographic approach that seeks to see the world from the vantage point of the patient allows an appreciation of the meaning and the experience of what might otherwise appear to be insignificant details. For example, in the vignette above Doris’s difficulties in washing her feet become symbolic of her social and physical situation. This mundane problem is at the centre of Doris’s world, but this centre does not hold steady (Strathern, 1991) when the perspective changes from zooming in on the individual experience to zooming out to system concerns – such as the cost effectiveness of a service. Instead it shifts to the side, becoming marginal. Moreover, given the temporal complexity referred to earlier in relation to changing narratives and experiences, the centre for an individual patient will not necessarily hold steady over time. Today’s burning concern will be de-centred when a new problem arises tomorrow, again on a seemingly trivial scale compared to the entrenched and long-standing problems that a health system has to address. These problems include an insufficient allocation of central government funding, and many years of being ‘under-doctored’. Representations of experiences developed from ‘close-up’ understandings of what matters to an individual are marginalised by the ‘wide angle’ view of what matters to a system. And yet, these facts are connected. This leads to the final tension I discuss here, that of perspective.

It is a matter of perspective that allows interventions to be brought into focus as distinct from ‘context’. Phenomenological and narrative interpretations of experience explain some of the differences between the perspective of ethnography and evaluation. A phenomenological understanding of a patient experience might not encompass a service intervention; an electronic care plan, for example, is simply not present in an embodied experience. An intervention might not be a ‘fact’ in the world of the patient, or it might not have left a lasting impression, instead having washed out, like a footprint in the sand (Hawe, Shiell, and Riley, 2009). To extend this metaphor, the immersed ethnographer can see the individual grains of sand, but in their proximity to the granularity of the social world, is unable to distinguish the bigger shape of the footprint. The time taken to develop a narrative understanding of patient experience also means that over time, the edges of the footprint will crumble, and the shape will blur and be lost, just as an intervention (and its impact) on someone’s life becomes indistinguishable from other events in their narrative. I am perhaps too close to be able to distinguish the effects of integrated care on research participants as distinct from other facts of their world.

## Unexamined epistemological differences

Ethnographic knowledge about patients’ experiences was produced from lengthy immersion and person-centred understanding, following what mattered to each person, and understanding their unique embodied experience within their specific social situation. Constructivist knowledge of this kind understands reality to be multiple, layered, shifting, embodied, and co-produced through interaction and language. I encountered no specific epistemological debates in the field; however, I did identify typical ways in which patients’ experiences were represented, revealing different assumptions about the nature of knowledge. In the field, patients’ experiences were represented in a number of formal, public texts, examples include; evaluation reports, consultation documents, and organisational Board papers. By recognising implicit assumptions about the purpose and nature of knowledge of patients’ experiences, tensions of size, scale and perspective experienced can be understood as epistemological differences. I describe below how representations of patients’ experiences are mobilised for specific purposes and the implications for epistemological conflicts with the knowledge produced from ethnography.

In formal consultation documents, patients’ experiences are typically used to illustrate, and endorse, proposed changes. Case studies are presented, with minimal, yet representative, personal details (name, age, living circumstances, health conditions), a chronology of interventions provided, and a third-person description of how the patient feels about the service. Patient stories articulate what otherwise might seem, to members of the public, vague or abstract proposals, illustrating in plain English how the services will be experienced and how they might matter to individuals. These stories, and short, direct quotes from patients, are also explicitly used to support proposed changes, chosen to fit into the overall argument for change. The use of patients’ experiences in this way is part of the ‘politics of participation’ (Pols, 2014). Stock photographs of patients and health care professionals are expected in this genre of consultation document, with short patient stories providing the individual, yet simultaneously representative, patient ‘voice’. The genre of Board meetings is a less usual setting for patient stories. One or two paragraphs describing interactions between individual local patients and services commissioned by the Boards are sometimes included in the lengthy meeting papers. The patient stories sit uncomfortably amidst the 200 and more pages of technical documents, next to inspection reports and ‘family and friends’ surveys. They are judged as either positive examples worthy of commendation, or negative examples that elicit explanation, and remedy. Negative stories require a response, an action that will address the problems identified; patients’ experiences are incorporated into a transaction, along the lines of ‘you said, we did’ – a trope of patient feedback and service response. Action, in the form of decision, is also the endpoint of evaluation.

Evaluation of the new integrated care service is being conducted to assess if it is meeting the aims of providing better patient experience and cost-effectiveness. An interim evaluation report provides further examples of how patients’ experiences are typically represented in the site. In this report, summaries of interviews with a sample of 10 patients and seven members of staff are presented. Patients’ views of the new service are illustrated with short quotes about their experiences (homely, welcoming, cups of tea are offered) and are compared with their generally less favourable views on their previous services. Staff members also report positive experiences and provide details of the efforts they make to improve patients’ experiences (holistic care, comfy chairs). Alongside the interview evidence, there is an analysis of cost-effectiveness undertaken by comparing more than one hundred patients registered with the service with a matched cohort of patients not registered with the service. There is discussion of the power required in the quantitative analysis to show ‘true change’. The juxtaposition of qualitative and quantitative data has troubling consequences, implying disconnection of patients’ experiences from outcomes, and reproducing the tensions of size and scale. Patients’ and staff experiences provide illustrative rather than explanatory details. The report is part of the process of quantifying and weighing the benefits of the intervention in order to take action, to make decisions about services. These decisions are necessarily made through a utilitarian framing of benefits, with primary concerns of cost effectiveness weighed against the ever-decreasing budget available for health services for the local population, an approach which makes ‘cups of tea’ and ‘comfy chairs’ seem rather trivial.

Patients’ experiences serve the purposes in the site of supporting particular arguments, of explaining and illustrating why changes might matter to patients, in conforming to expectations of genre, participation and voice, and providing evidence of experience. This latter evidence derived from patients, their experience of a new service, is presented as their subjective view. Patients’ experiences are therefore somewhat instrumental, added to the evaluation report alongside the ‘true’ change, and disembodied, separated from their narrative (Renedo, Komporozos-Athanasiou, and Marston, 2017). The instrumental nature of patients’ experiences in the site is in contrast to the instructive nature of ethnographic knowledge produced about patients’ embodied social worlds. The differences in purpose of knowledge relate to differences in the form of knowledge. In weighing up evidence for decisions, the experiences of ‘knowing subjects’ (Pols, 2005) are placed alongside quantitative evidence, resulting in the implicitly unfavourable comparisons of scale, robustness and relevance, and positioning these experiences as subjective facts about the (objective) world.

Instrumental use of patient experience renders it separable from the people and social situations where it originated, and stimulates a response. Patient experience becomes disembodied knowledge, a fact about the world to be weighed up with other facts, rather than providing instruction about the worlds of others. Facts remain about ‘cups of tea’ or ‘comfy chairs’ rather than being interpreted as facts about the meaning associated with being made comfortable, the lived experience of being made welcome or the narrative of being cared for by people that have time and resources to do so in a facilitative environment. Extracting facts from the narrative in which they occur, which is the required perspective of an evaluation, enables them to be seen as independent factors that can be addressed. Rather than understanding the meaning and precedents of particular events, action is stimulated to replicate, or prevent, them. The isolation of a specific event from its narrative and its lived experience allows it to be seen as preventable rather than intrinsically part of an embodied and emotional experience. Doris’s difficulties in washing her feet become small, insignificant facts about Doris, easily ignored, instead of being significant knowledge about the deep limitations, discomfort and isolation routinely experienced by this older couple despite being apparently well-housed and well-serviced by integrated care. Hospital admissions become seen as avoidable rather than the complex outcome of a series of events that are shaped by historic and geographical factors. Generalised approaches to isolated facts about the world provide a way of connecting a number of individual experiences, and stimulate action, but risk focusing on factors in isolation from the contexts which produce them. This means that efforts to address these factors might not be successful, as Hawe, Shiell, and Riley (2009) point out in relation to prevention activities which are based on individual psychological models rather than ecological models, and as can be seen in evaluation of integrated care programmes.

Facts are isolated from context, different kinds of evidence are presented in parallel; patients’ stories are buried in technical papers, summaries of interviews sit in parallel with quantitative analysis. Representations of patients’ experiences are not synthesised into new interpretations, instead they are acted upon, or not. In effect, the different kinds of knowledge are not socialised as there were few opportunities in the field for debate or dialogue. Without dialogue there is no opportunity to create the kind of hybrid knowledge Renedo, Komporozos-Athanasiou, and Marston (2017) describe in their research into public and patient involvement or opportunities for patients’ experiences to be made useful, transferable or turned into science (Pols 2014). Instead a phenomenological appreciation of patients’ experiences mingles uneasily with evaluations that, in their attempts to include patients’ perspectives, understand these to be multiple perspectives on an objective reality, knowable by measurement, rather than as different worlds, experienced uniquely. Without further discussion about the purpose and meaning of patients’ experiences, understandings of integrated care interventions remain system-focused rather than patient-focused.

## Concluding comments

My concern to understand the practice and experience of integrated care led to the development of ‘ways of knowing’ about the experiences of people with multiple long-term conditions from an appreciation of their facts about their world, their embodied situations, and their narratives. The process of generating and sharing phenomenological facts about the world and personal narratives was shaped by the temporal dynamics of fieldwork; elongating narratives and allowing re-interpretations. Tensions of size, scale and perspective occurred when trying to share knowledge of patients’ experiences, leading to an appreciation that although ethnography has the potential to explore how patient experience might be linked to service outcomes, this needs to be enacted. Dialogue and debate is needed, not just about what patients’ experiences are, but about the epistemological basis of these experiences – about what kind of knowledge they represent. Without this socialisation of knowledge of patients’ experiences, the reification of the large-scale de-centres and marginalizes lived experiences. These experiences, constituted of mundane daily lives and years past, weigh little in comparison with organisational priorities and government policy. Despite the potential for ethnography to add to evaluative processes, there is a risk that it remains impossible to reconcile very different understandings of the world without further dialogue about differences in the production, value and meaning of knowledge.

## Ethical approval

Ethical approval was gained from Camden and Islington Research Ethics Committee (13/LO/1610 29th November 2013).
